# A New Era of Image Guidance with Magnetic Resonance-guided Radiation Therapy for Abdominal and Thoracic Malignancies

**DOI:** 10.7759/cureus.2422

**Published:** 2018-04-04

**Authors:** Kathryn Mittauer, Bhudatt Paliwal, Patrick Hill, John E Bayouth, Mark W Geurts, Andrew M Baschnagel, Kristin A Bradley, Paul M Harari, Stephen Rosenberg, Jeffrey V Brower, Andrzej P Wojcieszynski, Craig Hullett, R A Bayliss, Zacariah E Labby, Michael F Bassetti

**Affiliations:** 1 Department of Human Oncology, University of Wisconsin - Madison, Madison, USA; 2 Radiation Oncology, Wentworth Douglas Hospital, Seacoast Cancer Center, Dover, USA; 3 Department of Radiation Oncology, University of Pennsylvania, Philadelphia, USA

**Keywords:** mr guided radiotherapy, on-line adaptive radiotherapy, gated tracking, real-time tracking, mri-guided adaptive radiotherapy

## Abstract

Magnetic resonance-guided radiation therapy (MRgRT) offers advantages for image guidance for radiotherapy treatments as compared to conventional computed tomography (CT)-based modalities. The superior soft tissue contrast of magnetic resonance (MR) enables an improved visualization of the gross tumor and adjacent normal tissues in the treatment of abdominal and thoracic malignancies. Online adaptive capabilities, coupled with advanced motion management of real-time tracking of the tumor, directly allow for high-precision inter-/intrafraction localization. The primary aim of this case series is to describe MR-based interventions for localizing targets not well-visualized with conventional image-guided technologies. The abdominal and thoracic sites of the lung, kidney, liver, and gastric targets are described to illustrate the technological advancement of MR-guidance in radiotherapy.

## Introduction

During the last two decades, remarkable progress has been made in the implementation of numerous imaging modalities for the simulation, planning, and delivery of radiation therapy. The early, prominent role of stand-alone computed tomography (CT) imaging systems has been enhanced by the onboard image-guidance technologies of portal imagers, orthogonal radiographic capabilities, megavoltage CT, optical tracking, and cone-beam CT (CBCT). Nonetheless, current CT-based image-guidance techniques still may result in suboptimal image-guidance capabilities due to insufficient soft-tissue contrast, motion degradation, surrogate-based tracking assumptions, and/or a lack of real-time tumor information. Magnetic resonance imaging (MRI) may allow for improvement upon prior CT-based image-guided radiotherapy (IGRT) capabilities. A new era in image-guided technology is rapidly evolving with the integration of an onboard magnetic resonance imaging (MRI) with a radiotherapy treatment system, with the emergence of magnetic resonance-guided radiation therapy (MRgRT).

In this case series, we illustrate unique capabilities of MRI guidance with a clinical MRgRT system (ViewRay MRIdian system, Oakwood Village, OH, US) for localizing targets not well-visualized by conventional IGRT techniques [1]. The abdominal and thoracic sites of the lung, kidney, liver, and gastric targets that were challenging to treat with CT-guided conformal intensity-modulated radiation therapy (IMRT) and stereotactic body radiation therapy (SBRT) are described.

## Case presentation

MRgRT workflow

**Figure 1:**
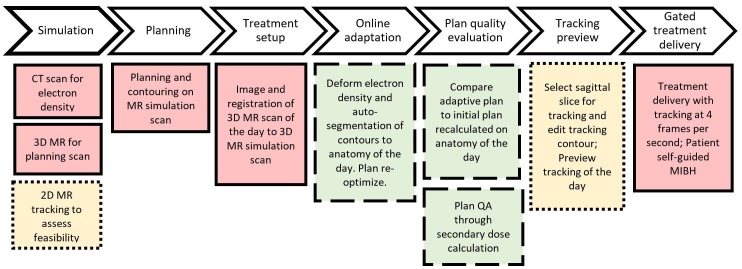
Clinical MRgRT workflow with workflow details unique to gating denoted in dotted outline and online adaptive in dashed outline. MR-guided radiation therapy (MRgRT); CT: computed tomography; MR: magnetic resonance; QA: quality assurance; MIBH: maximum inspiration breath-hold

The ViewRay MRIdian system is an integrated MRgRT system with onboard treatment planning and delivery capabilities. The MRIdian system combines a split-magnet 0.345 Tesla MRI design with a radiotherapy source of either cobalt-60 or six MV linac accelerator (LINAC) [1]. An overview of the MRgRT workflow utilizing the MRIdian cobalt-60 system at our institution is described here and shown in Figure 1. Note that workflow design will be institutional-specific and patient-specific, based on the treatment goals of a particular MRgRT program.

MR and CT simulation sessions are performed for anatomical guidance and electron density information, respectively. Simulation and delivery for a majority of MRgRT cases are performed under a comfortably moderate maximum inspiration breath-hold (MIBH). A balanced steady-state free precession sequence (TrueFISP) acquired in 17-25 second is utilized with a 1.5-mm in-plane resolution and 3.0-mm slice thickness [2]. During simulation, the patient is introduced to the “breathing sequence,” a self-guided MIBH sequence with free-breathing recovery. The patient is determined to be a candidate for MRgRT through MR screening and compliance with breath-hold (BH) instructions, claustrophobia, and tumor-tracking feasibility. In order to optimize the radiofrequency receiver coil's proximity to the patient’s surface and because delivery is performed with direct visualization under MR guidance, we have avoided external immobilization devices. Treatment planning and contouring are performed on MR simulation scan, with IMRT of nine to 12 beams. The electron density data from the CT scan is deformed to the planning MR scan at the time of plan generation. A Monte Carlo dose calculation is performed with magnetic field corrections.

For treatment delivery, initial patient localization is achieved through MR guidance, with the same 17-25 second MRIdian imaging protocol. When online adaptive radiotherapy is warranted, deformation of electron density and auto-segmentation are performed and evaluated, followed by plan re-optimization on the anatomy of the day. During treatment, the target is continuously tracked on an MR sagittal plane at four frames per second. The radiation beam is then gated based on the percentage volume of the tracking region of interest that strays beyond a pre-specified range.

Lung SBRT

A 67-year-old male, former smoker, was diagnosed with bilateral synchronous Stage I (T1a N0 M0) squamous cell carcinoma (SCC) of the left upper lobe (LUL) and Stage IA (T1a N0 M0) SCC of the right upper lobe (RUL). An 18F-fluorodeoxyglucose (18F-FDG) positron emission tomography (PET)/CT scan showed the LUL nodule was mild to moderately hypermetabolic with maximum standardized uptake values (SUV) of 4.0. The RUL was mildly hypermetabolic with a maximum SUV of 2.1. A biopsy was obtained from the LUL; it was positive for poorly differentiated SCC. A subsequent biopsy of the RUL cavitary lesion confirmed moderately differentiated SCC. MRI brain staging did not show metastases. These lesions were felt to be most consistent with two synchronous early stage lung cancers.

**Figure 2:**
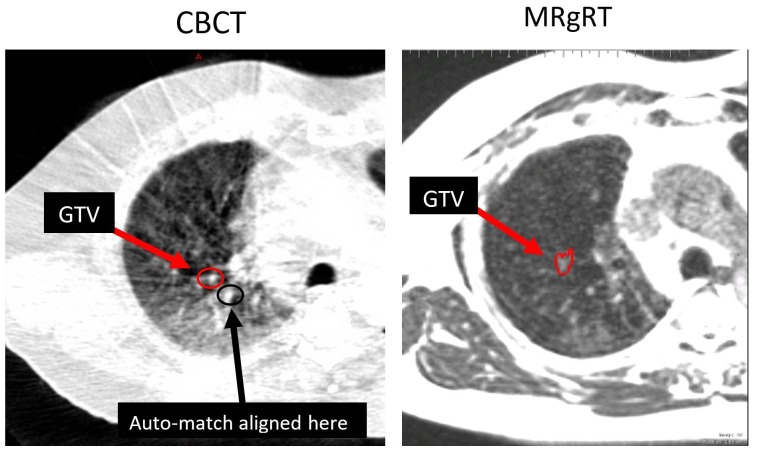
CBCT (left) and MRgRT scan (right). CBCT auto-match would have led to misalignment of GTV due to excessive noise of CBCT. CBCT: cone-beam computed tomography; MRgRT: magnetic resonance-guided radiotherapy; GTV: gross target volume

He was scheduled for SBRT of 50Gy in five fractions using CBCT-guidance on a conventional LINAC. A RUL gross target volume (GTV) measuring 0.89 cm^3^ was registered to the treatment planning CT simulation scan on the first fraction using CBCT localization (Figure 2). The auto-match CT-CBCT registration, utilizing a box-based rigid alignment around the planning target volume (PTV) resulted in a GTV misalignment of 12 mm. Upon CT-CBCT registration review, the attending physician decided to abort CBCT-guided treatment and transfer the patient to the MRIdian system to minimize the potential of a geometric miss. The RUL GTV was well-visualized on the 25-second 3D MR setup scan acquired at MIBH (Figure 2), minimizing motion artifacts.

For MR-guided SBRT, the RUL GTV was defined as the tracking structure with a 5.0-mm margin expansion delineating the gating boundary. Tracking was performed at four frames per second with the treatment beam turned off when ≥10% of the GTV exceeded the gating boundary. Gating latency for the ViewRay MRIdian cobalt-60 system includes image frequency (250 msec), source shuttling, and processing time and has been reported at 436 msec [3]. The dosimetric impact of gating latency has been reduced through the utilization of BH, i.e., reducing the overall number of beam on/off transitions.

**Figure 3:**
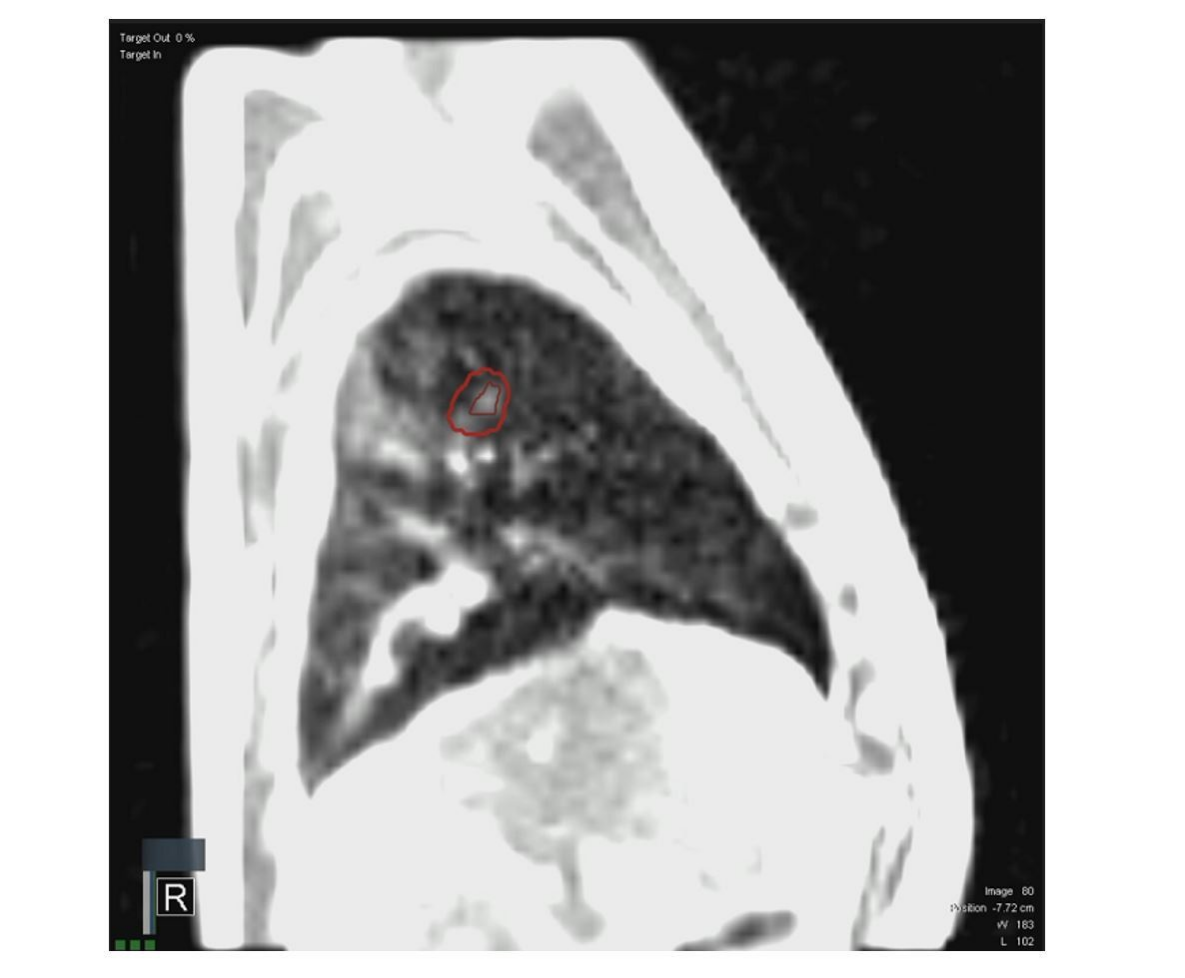
Real-time tracking of 0.89 cm3 gross target volume with 5.0 mm boundary was feasible on clinical magnetic resonance-guided radiotherapy system, minimizing potential geometric miss observed from auto-match of cone-beam computed tomography-based registration for patient. ​​​​​​​

The deform-generated GTV (i.e., tracking structure) was visually inspected during the pretreatment preview cine, in addition to the real-time cine acquired during treatment delivery. Even with a limited in-plane resolution of 3.5 mm for sagittal plane tracking, the deformable image registration (DIR) algorithm appropriately identified and segmented the GTV, as observed with visual inspection (Figure 3). Treatment was well- tolerated with minimal side effects. At 16 months' post-follow-up, the patient continues to feel well. Chest CT shows the expected changes of radiation-induced lung injury without evidence of recurrent or metastatic intrathoracic disease.

Kidney SBRT

A 60-year-old man initially presented with stage III (T2N1M0) SCC of the right tonsil. He underwent transoral robotic surgery of the right tonsil and level II-IV selective lymph node dissection with one of 25 lymph nodes involved with extra-capsular extension and adjuvant 66Gy in 22 fractions radiation with concurrent weekly cisplatin (40 mg/m2) to the primary and bilateral neck. One year later, he presented with two oligometastatic lung lesions. He initiated cisplatin and 5-fluorouracil. Cisplatin was transferred to carboplatin due to peripheral neuropathy. After disease progression with carboplatin and docetaxel, SBRT was treated to the two lesions. Seven months later a PET/CT showed a new intense focal 18F-FDG uptake near the right hilum associated with a new 2.3x1.8 cm metastatic malignancy. The PET scan revealed hyperintense metabolic activity in the superior pole of the right kidney, measuring 4.2x4.4 cm. A biopsy of the renal mass confirmed metastasis SCC. The renal function was 43.1% (right kidney) and 56.9% (left kidney) with a creatinine of 1.17 (estimated glomerular filtration rate = 70).

**Figure 4:**
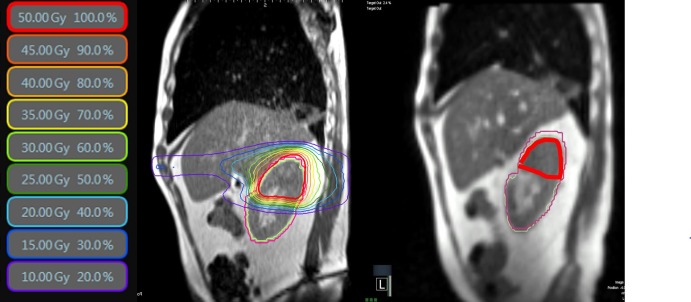
Treatment plan for stereotactic body radiation therapy to the kidney (left). Treatment delivery magnetic resonance cine frame with real-time tracking of kidney at four frames per second to gate treatment beam on/off (right).

Respiratory motion for potential internal target volume (ITV)-based treatment with conventional CT-based IGRT was assessed using four-dimensional (4DCT) with abdominal compression; kidney motion up to 0.75 cm along the cranial-caudal direction was observed under shallow breathing. With excellent control of the previously treated lung sites and limited treatment options, we planned treatment to both sites with MRI-guided SBRT utilizing MR-guided gating under MIBH. Treatment was planned for 40Gy in five fractions to the hilar mass. A treatment course to the superior right kidney while sparing the uninvolved lower right kidney was planned for 50Gy in five fractions (Figure 4, left). Given the high degree of contrast between the kidney and abdominal fat, the entire kidney was tracked (though treatment was only to the superior pole) with radiation trigged when ≥98% of the entire kidney was within the 3.0-mm boundary expansion, as illustrated in Figure 4, right.

**Figure 5:**
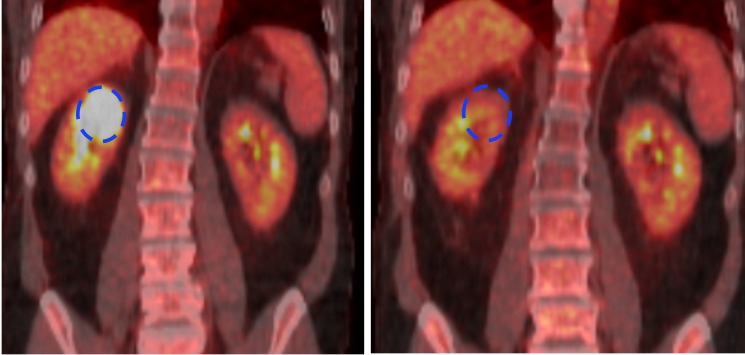
Pre-treatment PET (left) and post-treatment PET (right) for stereotactic body radiation therapy to the kidney treated on MRIdian system. PET: positron emission tomography

Treatment was initiated two weeks after the biopsy. Following the first fraction, the patient developed pain and nausea and passed a blood clot in his urine, likely due to a post-biopsy-related clot although the contribution of SBRT was not completely excluded. The pain was managed with morphine and treatment to the lung continued. Treatment to the kidney was initially held and resumed two weeks later. The patient tolerated the remaining treatment well with only moderate fatigue. Follow-up PET/CT imaging showed excellent local control. This case illustrates the ability of MRgRT to deliver highly conformal and safe SBRT to the involved superior pole while sparing the uninvolved portion of the ipsilateral kidney. The hypermetabolic right kidney lesion was no longer 18F-FDG avid in the post-treatment scan, with normal metabolism visible in the lower portion of the treated kidney (Figure 5).

Liver SBRT

A 79-year-old man had an incidentally discovered 5.2-cm mass in the dome of the liver. A biopsy revealed well-differentiated hepatocellular carcinoma (HCC) and the patient was initially planned to undergo transarterial chemoembolization. Pulmonary function testing revealed anesthesia as a significant risk. He was, therefore, planned to undergo SBRT to the liver GTV. The patient was scheduled for 55Gy in five fractions of MR-guidance; however, due to non-patient-related circumstances, he was transferred to CBCT-based LINAC for the remaining four fractions. This case was unique, as it allowed a direct comparison of onboard image guidance with MRI vs CBCT.

**Figure 6:**
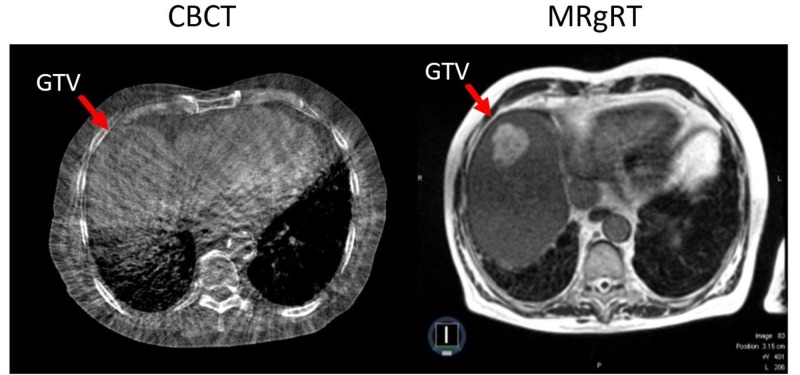
GTV localized with CBCT (left) and MRIdian MRgRT setup scan (right) for fraction 2 and fraction 1, respectively, for patients undergoing liver stereotactic body radiation therapy for hepatocellular carcinoma. GTV: gross target volume; CBCT: cone-beam CT; MRgRT: magnetic resonance-guided radiation therapy

Since treatment was performed with CBCT-based localization for four of five fractions, case details are limited to fraction 1 delivery with MRgRT. Gadoxetate has been demonstrated to be well-tolerated in MRgRT for repeated use over an SBRT liver course for tumor localization and real-time tracking [4] but is not generally helpful for primary HCC. This HCC was well visualized under MR-guidance (Figure 6), compared to the CBCT.

**Figure 7:**
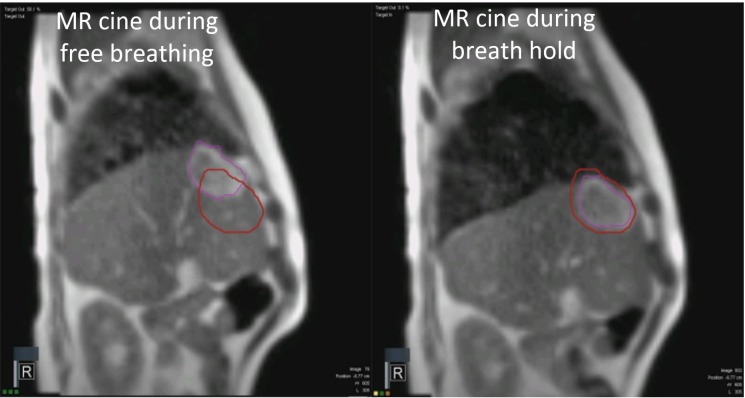
Direct visualization of hepatocellular carcinoma in MR-gated delivery. Beam off (left) during tumor out of treatment field, and beam on (right) during tumor within the treatment field at breath-hold. MR: magnetic resonance

The GTV was assigned as the tracking structure for MIBH MR-gated delivery (Figure 7). When ≥10% of the tracking volume exceeded the boundary volume (3.0-mm uniform expansion), the beam was automatically turned off. The utilization of MR-guided gated MIBH allowed for a reduction of the PTV margin (3.0-mm GTV to PTV margin) for MRgRT compared to the CBCT-based plan, delivered under shallow breathing with abdominal compression. For CBCT-based treatment, a 3.0-mm ITV to the PTV margin was used. GTV changes (incorporated within the internal margin) with respiratory motion during 4DCT with abdominal compression measured up to 1.2 cm along the cranial-caudal and anterior-posterior directions.

Gastric lymphoma

A 70-year-old man with stage IIAE-X gastric diffuse large B-cell lymphoma, germinal center type International Prognostic Index 3.0 underwent esophagogastroduodenoscopy and biopsy, demonstrating a gastric mass in the body of the stomach, with the biopsy showing diffuse large B-cell lymphoma, germinal center type. The PET scan demonstrated a marked hypermetabolic soft tissue conglomerate, diffuse thickening of the stomach wall extending to the first portion of the duodenum, and multiple hypermetabolic soft tissue deposits adjacent to the stomach, consistent with disease extension. The patient received six cycles of rituximab plus cyclophosphamide, doxorubicin, vincristine, and prednisone (R-CHOP) chemotherapy, with an interim PET scan after cycle 5 read as Deauville 4.0, nonspecific for the residual lymphoma versus component of post-chemotherapy inflammation with biopsies demonstrating inflammation.

**Figure 8:**
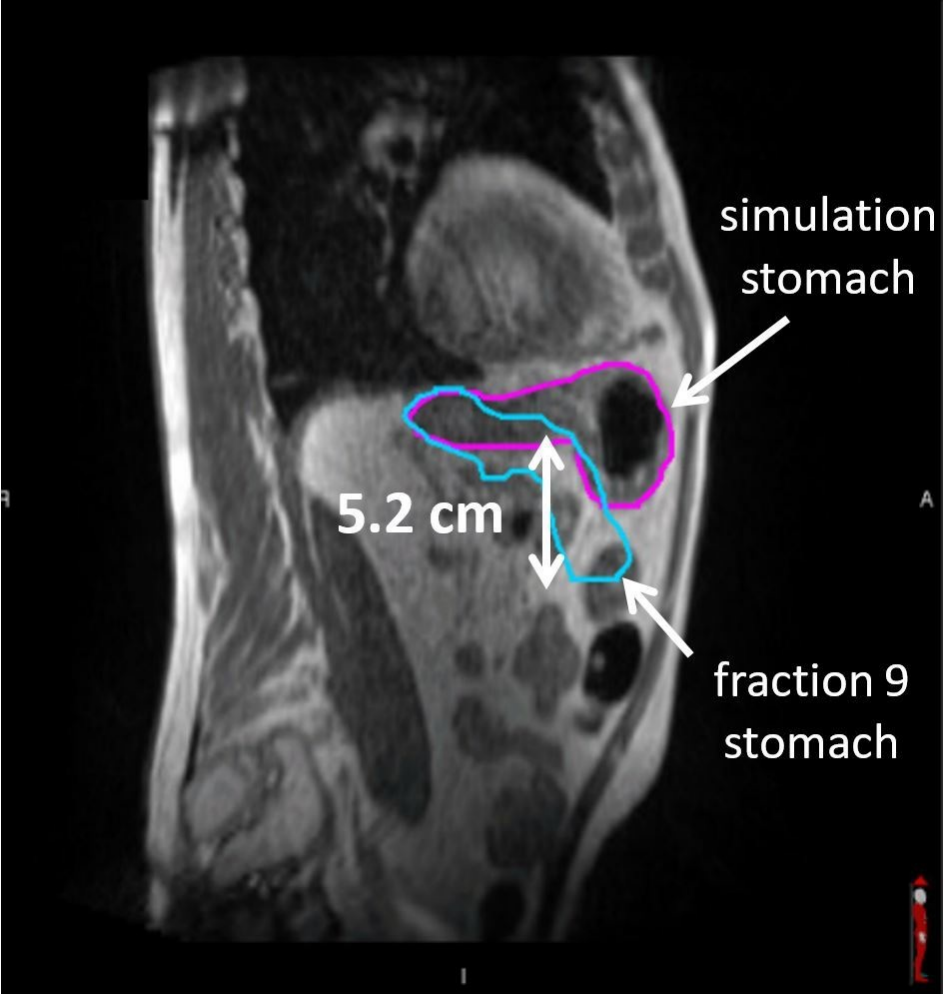
Large inter-fraction stomach deformation between magnetic resonance simulation to fraction 9, for the case with the patient compliant with no eating or drinking four hours to radiotherapy.

Consolidative involved site radiotherapy to the stomach and perigastric lymph nodes was recommended, and 30Gy in 20 fractions was delivered using MRI-guided IMRT. The patient was instructed to not eat or drink starting four hours prior to treatment. Significant inter-fractional stomach variations on the order of 5.0 cm were observed (Figure 8).

**Figure 9:**
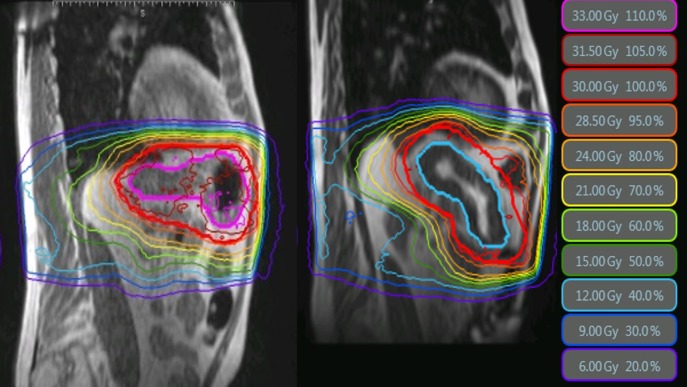
Initial plan based on simulation magnetic resonance scan (left) and magnetic resonance-guided adapted plan (right) for fraction 9 for gastric lymphoma.

Over the course of MRgRT, large deformation and inter-fraction variations were systematically observed between two geometries, as shown in Figure 9. The stomach deformation across 20 fractions quantified by Dice similarity coefficient (DSC) was 0.58±0.19, with fraction 9 at 0.38 DSC (Figure 8). A plan-of-the-day approach was used to efficiently adapt based on the stomach geometry. MR-guided gating was performed with beam off when ≥10% of the stomach volume exceeded the 3.0-mm boundary expansion. The MRgRT course was well-tolerated. There is no clinical evidence of residual or recurrent disease currently at 28 months post-treatment.

## Discussion

MR-guidance represents a fundamental shift in IGRT utility and methodology. Prior IGRT technologies are often based on the philosophy of daily matching the patient to a prior snapshot of anatomy (i.e., simulation CT), requiring substantial and variably effective immobilization. MRgRT allows for the ability to dynamically adjust to the patient’s anatomy of the day by tailoring changes in dose distributions and tumor tracking.

The quality and accuracy of online adaptation and real-time tracking rely on the performance of the DIR algorithm. Deformation and dosimetric accuracy for an MR-guided online adaptive radiotherapy program have been evaluated with a deformable, anthropomorphic phantom [5]. The mean dose differences of calculated to measured implanted dosimeters were within clinical trial dosimetric criteria for phantom validation. The mean DSC of online adaptive auto-segmentation was 0.8 (MR-MR deformation) and electron density propagation was 0.9 (CT-MR deformation) for the deformable phantom.

The dosimetric effects of a gating latency of 436 msec [3] have been mitigated for the cases presented using a breath-hold approach. The median breath-hold duration for patients under MRgRT at our institution has been observed at 26 sec, allowing <2% latency error. Further reduction of gating latency for MR-linac design is anticipated. The dosimetric accuracy of gated MRgRT delivery was investigated by Lamb et al. and found to be within 97.8% for 3%/3mm gamma criteria on the MRIdian cobalt-60 system using a 3-mm GTV to boundary margin and a 3.5-mm in-plane cine resolution [3].

While such daily precision and personalized medicine of MRgRT may not be warranted for all anatomical sites of radiotherapy, the role of MR-guidance in the SBRT of the thoracic and abdominal sites offers clear advantages, as demonstrated by the cases presented here. Prior reporting of the clinical utilization of MRgRT has been limited in the literature to abdominal areas [4,6-7] or review articles of potential advantages for pelvis [8] and thoracic [9] sites. Experiences of the clinical utility of MR-gating capabilities have also been limited [10]. This case study represents a comprehensive series of the abdominal and thoracic utilization of MRgRT, with a direct comparison of CBCT localization for a subset of cases. For all cases presented, increased confidence in superior target coverage and avoidance of a potential miss-treatment were achieved through 3D MR-guided localization, MR-guided gating, and MR-guided online adaptation. 

MRgRT has great promise to allow for the safe delivery of a higher biologically effective dose to the treatment area that may not have been feasible with prior technology. Adaptive radiotherapy allows for dose escalation to a target when the neighboring organ at risk (OAR) has moved away from the treatment area or for dose de-escalation to safely achieve normal tissue toxicity constraints when OAR has moved into the treatment area. The new era of MR guidance may enable changes to clinical practice, including a higher dose per fraction, reduced treatment margins with potentially reduced toxicity, and a lower likelihood of geometric miss. Clinical trials will be necessary to fully understand the potential benefits of this technology.

## Conclusions

MR guidance for thoracic and abdominal tumors of the lung, liver, stomach, and kidney is feasible and allows for improved localization over conventional CT-based IGRT capabilities. Superior soft-tissue visualization combined with the MRgRT ability to dynamically adjust the treatment plan and/or gate the treatment delivery to account for inter-/intrafractional anatomical changes offers great promise to further enhance treatment precision for abdominal and thoracic anatomical sites. This approach brings valuable opportunities to decrease overall toxicity profiles.
